# Circulating H3K27 Methylated Nucleosome Plasma Concentration: Synergistic Information with Circulating Tumor DNA Molecular Profiling

**DOI:** 10.3390/biom13081255

**Published:** 2023-08-16

**Authors:** Emmanuel Grolleau, Julie Candiracci, Gaelle Lescuyer, David Barthelemy, Nazim Benzerdjeb, Christine Haon, Florence Geiguer, Margaux Raffin, Nathalie Hardat, Julie Balandier, Rémi Rabeuf, Lara Chalabreysse, Anne-Sophie Wozny, Guillaume Rommelaere, Claire Rodriguez-Lafrasse, Fabien Subtil, Sébastien Couraud, Marielle Herzog, Lea Payen-Gay

**Affiliations:** 1 Center for Innovation in Cancerology of Lyon (CICLY) EA 3738, Faculty of Medicine and Maieutic Lyon Sud, Claude Bernard University Lyon I, 69921 Oullins, France; 2 Pulmonology Department, Lyon Sud Hospital, Hospices Civils de Lyon, 69495 Pierre-Bénite, France; 3 Belgian Volition SRL, Parc Scientifique Créalys, 5032 Isnes, Belgium; 4 Institute of Pharmaceutical and Biological Sciences (ISPB), Claude Bernard University Lyon I, 69373 Lyon, France; 5 Department of Biochemistry and Molecular Biology, Lyon-Sud Hospital, Hospices Civils de Lyon, 69495 Pierre-Bénite, France; 6 Circulating Cancer (CIRCAN) Program, Hospices Civils de Lyon, Cancer Institute, 69495 Pierre-Bénite, France; 7 Pathology Department, Claude Bernard University Lyon I, Hospices Civils de Lyon, 69677 Bron, France; 8 Cellular and Molecular Radiobiology Laboratory UMR CNRS5822/IP2I, Faculty of Medicine and Maieutic Lyon Sud, Claude Bernard University Lyon I, 69921 Oullins, France; 9 Statistic Department, Hospices Civils de Lyon, 69008 Lyon, France; 10 LBBE, Claude Bernard University Lyon I, UMR 5558, CNRS, 69100 Villeurbanne, France

**Keywords:** NSCLC, epigenetics, histone PTM, H3K27Me3 nucleosome, ctDNA, liquid biopsy, biomarkers, MRD

## Abstract

The molecular profiling of circulating tumor DNA (ctDNA) is a helpful tool not only in cancer treatment, but also in the early detection of relapse. However, the clinical interpretation of a ctDNA negative result remains challenging. The characterization of circulating nucleosomes (carrying cell-free DNA) and associated epigenetic modifications (playing a key role in the tumorigenesis of different cancers) may provide useful information for patient management, by supporting the contributive value of ctDNA molecular profiling. Significantly elevated concentrations of H3K27Me3 nucleosomes were found in plasmas at the diagnosis, and during the follow-up, of NSCLC patients, compared to healthy donors (*p*-value < 0.0001). By combining the H3K27Me3 level and the ctDNA molecular profile, we found that 25.5% of the patients had H3K27Me3 levels above the cut off, and no somatic alteration was detected at diagnosis. This strongly supports the presence of non-mutated ctDNA in the corresponding plasma. During the patient follow-up, a high H3K27Me3-nucleosome level was found in 15.1% of the sample, despite no somatic mutations being detected, allowing the identification of disease progression from 43.1% to 58.2% over molecular profiling alone. Measuring H3K27Me3-nucleosome levels in combination with ctDNA molecular profiling may improve confidence in the negative molecular result for cfDNA in lung cancer at diagnosis, and may also be a promising biomarker for molecular residual disease (MRD) monitoring, during and/or after treatment.

## 1. Introduction

Nucleosomes are small fragments of chromosomes released into the blood during cell death, and they consist of a histone octamer core with DNA wrapped around it. Interestingly, the size distribution of the DNA released into circulation (cell-free DNA (cfDNA)) corresponds to nucleosomes (+/−147 bp) or chromatosomes (nucleosome + DNA linker; +/−167 bp) [1]. CfDNA is released into the bloodstream via various mechanisms, most predominantly cell-death mechanisms, although other forms of release could exist [2]. CfDNA can circulate in a nucleosomal format, or in other DNA–protein complexes, as well as encapsulated in extracellular vesicles, which protect it from rapid degradation by nucleases [3,4]. In cancer patients, cfDNA/nucleosomes originate from multiple sources, including malignant cells, cells from the tumor microenvironment, or non-cancer cells [5]. On one hand, a portion of the cfDNA released may be derived from tumor cells (circulating tumor DNA (ctDNA)), and may contain mutations. On the other hand, nucleosomes could be also released from cancer cells, and may harbor a variety of histone post-translational modifications (PTMs) [6]. Higher levels of cfDNA and nucleosomes were found in cancer samples, compared to healthy samples [3,7,8].

Histone protein PTMs, such as methylation, acetylation, phosphorylation, or citrullination regulate most of the DNA-templated processes, including replication, transcription, and repair. They function as platforms for the recruitment of specific effector proteins, such as transcriptional regulators or chromatin remodelers [9]. They can also change the chromatin structure by disrupting the electrical charges in the histone residues and, thus, modulate the accessibility of the DNA [10,11]. Histone methylation is dynamically controlled by various lysine histone demethylase (HDM) and histone methyltransferase (HMT) enzymes that remove or add the methyl group(s) from the lysine residues. The mis-regulation of HDM and HMT leads to aberrant levels of histone methylation, and has been associated with a variety of cancer types, including breast, prostate, lung, and brain cancers [6]. For example, the HMT enzyme, enhancer of zeste homolog (EZH2), the catalytic subunit of polycomb repressive complex 2 (PRC2), responsible for the trimethylation of histone H3 at lysine 27 (H3K27Me3), has been reported to be upregulated in cancer [12,13]. Its overexpression also correlates with breast cancer aggressiveness, and poor prognosis [14]. In parallel, H3K27Me3 levels are associated with transcriptional repression, and have been reported to play an important role in the development and progression of several cancers, including lung cancer [15,16,17]. EZH2-catalyzed H3K27Me3 is also a master regulator of the epithelial mesenchymal transition (EMT), an early cellular reprogramming event to facilitate the initial steps of the metastatic cascade [18,19,20]. EZH2 levels are impaired in tumor angiogenesis, and are associated with a drug-resistance phenotype in multiple cancers [21,22].

Epidermal growth factor receptor (EGFR)-tyrosine kinase inhibitors (TKIs), such as osimertinib and gefitinib, have only been proven to be beneficial for patients with EGFR-sensitizing genetic alterations in lung cancer [23]. Recently, it had been shown that EZH2 inhibitors could sensitize in vitro EGFR wild-type cells to gefitinib [24]. EZH2 is highly expressed in non-small cell lung cancer (NSCLC), and is associated with poor prognosis in patients with lung adenocarcinomas. Therefore, targeting H3K27Me3 via EZH2 is a promising cancer therapy. Tazemestostat is the only EZH2 inhibitor approved by the FDA for adults and pediatric patients aged 16 years and older. Currently, there are several ongoing clinical trials of drugs targeting EZH2 in different cancer types [25].

Combined ctDNA molecular testing, to analyze circulating nucleosomes and their histone PTMs before and after patient treatment, could provide a wider view of the genetic and the epigenetic landscape of a tumor, and its response to treatment. This would also enable a minimal residual disease (MRD) assessment. Therefore, we conducted this retrospective, non-interventional study to (i) determine, at diagnosis, the level of circulating nucleosomes containing methylated histone residues, especially H3K27Me3 nucleosomes as potential biomarkers in patients with stage IV NSCLC; and (ii) assess, during treatment, the levels of H3K27Me3 nucleosomes in NSCLC with a different mutated ctDNA status, to evaluate MRD in driving the choice of the following treatment line. We studied the ctDNA analysis association with nucleosome biomarkers in two independent cohorts of NSCLC patients: the first at diagnosis, before any treatment; and a second undergoing treatment, including chemotherapy, combined or not with immunotherapy, or targeted therapy. The nucleosome measurements were performed on the same sample as the molecular ctDNA testing in a routine setting. This strengthens the accuracy of using biomarkers in patient management of NSCLC.

For the first time, we report a high level of circulating H3K27Me3 nucleosomes in NSCLC samples compared to healthy samples, and the potential benefit of the combination of information generated by the measurement of H3K27Me3 nucleosome levels, in association with the molecular ctDNA profile at diagnosis, in patient management. We also highlight the putative role of circulating H3K27Me3 nucleosomes as blood-based biomarkers in quantifying the MRD, to monitor NSCLC patients during treatment.

## 2. Materials and Methods

### 2.1. Study Population

Patients were recruited from the pulmonology department at Lyon University Hospital from 2015 to 2022. K2-EDTA plasma heathy samples were provided by the French agency named “Etablissement Français du Sang” (Etude RNIPH 22-5065 NUCLEO_CIRCAN avis CSE n° 22-5065). Inclusion criteria were: aged over 18 years old, with histologically proven NSCLC. For each sample, medical data were collected through a mandatory prescription sheet attached to each sample, and edited by the prescribing physician. K2-EDTA plasma samples from 319 patients at diagnosis, and from 304 independent patients under treatment, defined via a clinical or CT-scan modification during their treatment, were analyzed.

### 2.2. CfDNA Collection

The total EDTA blood samples were centrifuged for 10 min at 1600× *g*. The supernatant was then centrifuged at 6000× *g* for 10 min, and the resulting plasma was stored at −80 °C, until the cfDNA extraction and molecular analysis was conducted [26]. CfDNA was extracted using the QIAamp Circulating Nucleic Acid Kit (Qiagen, Valencia, CA, USA, Cat No 55114), with a Qiagen vacuum manifold, following the manufacturer’s instructions. CfDNA samples were quantified using a Qubit™ 4 Fluorometer (Invitrogen™, Cat No Q33238, Carlsbad, CA 92008, USA), with the Qubit™ dsDNA HS Assay Kit (Invitrogen™, Cat No 32854).

### 2.3. Library Preparation for DNA Sequencing

For the custom-validated NGS library preparation, 10–100 ng cfDNA were used, using a custom capture-based technology provided by SOPHiA GENECTICS (Lausanne, Switzerland), and performed according to the manufacturer’s instructions [27,28,29]. The custom panel covered 78 genes involved in cancer (such as *EGFR*, *TP53* or *KRAS*, see Supplemental Information—Supporting Information S1—for the entire list of genes). The libraries were sequenced on NextSeq 550 (Illumina Technology, San Diego, CA, USA) in 2 × 150 paired-end runs. The subsequent variant call files were subjected to cross-sample background filtering, with potential artefacts removed below three standard deviations of the mean background noise for each position. The bioinformatics was performed using the SOPHIA DDM^TM^ platform.

### 2.4. Nu.Q^®^ ImmunoAssays

Four nucleosome structures were measured using Nu.Q^®^ prototype immunoassays: Nu.Q^®^ H3K27Me3, Nu.Q^®^ H3K36Me3, Nu.Q^®^ H3K9Me3, and Nu.Q^®^ H3K4Me2 (Belgian Volition SRL, Isnes, Belgium), according to the manufacturer’s instructions. Briefly, these sandwich immunoassays are based on magnetic bead and chemiluminescence technology, and are performed using the IDS-i10 automated immunoanalyzer system (Immunodiagnostic Systems Ltd. (IDS), Boldon, UK). An amount of 50 μL of K2-EDTA plasma (the same as for the DNA sequencing) is incubated with acridinium ester labeled as the anti-nucleosome antibody. Then, magnetic particle beads, coated with the corresponding monoclonal anti-histone modification capture antibody (i.e., anti-histone H3K27Me3, anti-histone H3K36Me3, anti-histone H3K9Me3, or anti-histone H3K4Me2, respectively), are added. Finally, after a wash step, trigger solutions are added, and the light emitted by the acridinium ester is measured via the luminometer system. The results are expressed in relative light unit (RLU), and the concentrations are extrapolated using the four-parameter logistic regression of a reference standard curve. All samples are analyzed in singlicate.

### 2.5. Tissue Microarray (TMA)

Two TMAs were purchased from the company TissueArray.com. The first TMA corresponds to 100 cores of 50 cases (two cores by case) of lung adenocarcinoma (BC04022a), while the second TMA contains 18 cases of squamous cell carcinoma, matched with the adjacent normal lung tissue and cancer-adjacent tissue per case (LC10014a).

The immunohistochemical analysis was performed using an automated immunostainer (BOND). Briefly, formalin-fixed paraffin-embedded 4-μm-thick sections were, firstly, paraffinized in xylene, and rehydrated in ethanol. The endogenous peroxidase activity was blocked (Ventana Medical Systems, Tucson, AZ, USA) before antigen retrieval was commenced. The ULTRA Cell Conditioning Solution (ULTRA CC1) from Ventana Medical Systems was then used for antigen retrieval. Immunohistochemical staining was carried out using an automated immunostainer, with a primary antibody. This was followed by the application of the avidin–biotin–peroxidase complex technique. Reactions were developed using diamino-3,3′-benzidine tetrahydrochloride substrate solution (SIGMAFAST; Sigma-Aldrich, Tucson, AZ, USA). The tissues were then counterstained with hematoxylin. The primary antibodies and final dilutions were: H3K27Me3 (1/500) (Cell Signaling Technology, Danvers, MA, USA).

Immunostaining in the tumor tissue was determined via visual scoring of the brown stain. Scoring of the intensity of the staining was performed according to an arbitrary scale with steps of 0, 1, 2, and 3, where “0” was considered to be the absence of staining, “1” was considered weak staining, “2” was considered as moderately positive staining, and “3” was considered to be strong staining.

### 2.6. Statistics

Statistical analyses were performed using GraphPad Prism (GraphPad Prism software version 9.5.0, San Diego, CA, USA). Descriptive statistics were used, and results are reported as the mean, median, 25th, and 75th percentiles. The data were subjected to the Kolmogorov–Smirnov normality test. As the data are not normally distributed, non-parametric methods were used: the Mann–Whitney U test and Kruskal–Wallis H test for group comparison, and Spearman’s rank correlation for measuring the dependence between variables. Significance values are represented by *: *p*-value < 0.05; **: *p* < 0.01; ***: *p* < 0.001; ****: *p* < 0.0001. The reference interval (also referred to as the normal range) of the H3K27Me3-nucleosome levels was calculated on a global healthy cohort, to reach the statistically significant minimum number (*n* ≤ 120) defined in the CLSI guideline EP28-A3c. The data were subjected to D/R ratio and ROUT (robust regression and outlier removal) methods for the detection of outliers. The D/R ratio method was performed as described in the CLSI guideline EP28-A3c. The ROUT method was performed using GraphPad Prism software; the coefficient Q was set at 1%, as recommended. The reference interval includes 95% of the population, from the 2.5th to 97.5th percentile. One standard deviation was added to the upper limit of this reference interval, to determine the cut off used to define low and high H3K27Me3-nucleosome (referred as H3K27Me3-negative and -positive, respectively) levels in the decision tree model proposed.

### 2.7. Study Limitations

As this was a retrospective study carried out for routine use, we have faced limitations, including: (i) the access to clinical data is limited to the ctDNA molecular profile and the time of sampling in routine patient management; (ii) we did not have matched tissue–plasma samples, so the histological analyses were performed using a commercial tissue macro-array; (iii) independent cohorts were analyzed at diagnosis and during treatment; (iv) patients were not followed at multiple time points, and the disease progression was not associated with the imaging; and (v) the levels of circulating nucleosomes containing specific histone methylation marks were measured independently for each mark, and reported as an absolute quantity of H3K27Me3-, H3K36Me3-, H3K9Me3, or H3K4Me2-nucleosomes.

## 3. Results

### 3.1. High Levels of Circulating H3K27Me3 Nucleosomes Are Observed in NSCLC Samples at Diagnosis

**Figure 1:**
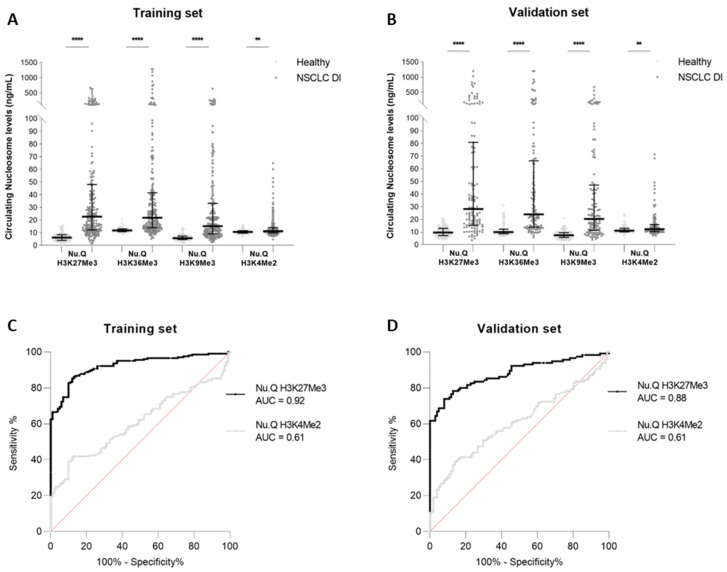
Quantification of circulating methylated nucleosomes in NSCLC samples at diagnosis, and in healthy samples. The concentration of circulating H3K27Me3, H3K36Me3, H3K9Me3, or H3K4Me2 nucleosomes was measured, using chemiluminescent Nu.Q^®^ immunoassays, in human K2-EDTA plasma samples from NSCLC patients at diagnosis (DI) and from healthy donors. (**A**,**B**) Jitter plot analysis representations of the circulating H3K27Me3, H3K36Me3, H3K9Me3, and H3K4Me2 nucleosome levels in NSCLC samples, compared to healthy samples, in the training (**A**) and validation (**B**) sets. The whiskers represent the 25th–75th percentile with median. ** and **** represent *p*-value < 0.01 and < 0.0001, respectively, calculated using the Mann–Whitney U test. (**C**,**D**) Receiver-operating characteristic (ROC) curve analysis of the circulating H3K27Me3 and H3K4Me2 nucleosomes for the discrimination of NSCLC at diagnosis, versus healthy samples, in the training (**C**) and validation sets (**D**). The areas under the curve (AUC) for the targeted nucleosome markers are indicated in the legend. The red line indicates the theoretical random chance. The corresponding significance *p*-values for the circulating H3K27Me3 and H3K4Me2 nucleosomes are, respectively, <0.0001 and 0.0021 for the training set, and *p* < 0.0001 and *p* = 0.0063 for the validation set.

The concentration of circulating nucleosomes with the specific methylated marks H3K27Me3-, H3K36Me3-, H3K9Me3-, and H3K4Me2- were measured using chemiluminescent Nu.Q^®^ immunoassays for the NSCLC samples (training set (T) n_T_ = 203; validation set (V) n_V_ = 116), and were compared to healthy samples (n_T_ = 100; n_V_ = 101). In both sets, we observed significantly higher circulating H3K27Me3-, H3K36Me3-, or H3K9Me3-nucleosome levels in NSCLC samples compared to the healthy ones (median_T_: 22.7 ng/mL vs. 6.1 ng/mL; 21.8 ng/mL vs. 11.8 ng/mL; 15.2 ng/mL vs. 5.7 ng/mL, respectively; *p*-value < 0.0001 for each and median_V_: 28.3 ng/mL vs. 9.6 ng/mL; 24.0 ng/mL vs. 10.0 ng/mL; 20.4 ng/mL vs. 7.5 ng/mL, respectively; *p*-value < 0.0001 for each) (Figure 1A,B). A lower difference was observed in H3K4Me2-nucleosome levels between the NSCLC and healthy samples (median_T_: 11.3 ng/mL vs. 10.6 ng/mL and median_V_ = 12.4 ng/mL vs. 11.2 ng/mL, respectively; *p*-value < 0.01 for each), with a very small magnitude (Table S1). The highest change observed between the medians of the cancer and healthy samples was observed in the level of H3K27Me3 nucleosomes, with a fold change of 3.7 and 2.9 in the training and validation sets, respectively (Table S1). This marker also showed the best area under the ROC (receiving operating characteristic) curve (AUC), with a value of 0.92 (95% CI: 0.89–0.95) and 0.88 (95% CI: 0.84–0.93), corresponding to the training and validation set analysis (Figure 1C,D and Figure S1A,B). The sensitivity of the H3K27Me3 assay reached 69.5% (95% CI: 61.8–74.5%) at 95% specificity in the training set. These clinical performances were confirmed in the validation set, where the sensitivity reached 68.7% (95% IC: 59.7–76.5%) at 95% specificity (Figure 1C,D and Table S2A,B).

### 3.2. H3K27Me3-Nucleosome Levels Correlate with cfDNA Quantity and Percentage of Mutational Allele Fraction (MAF) at Diagnosis

**Figure 2:**
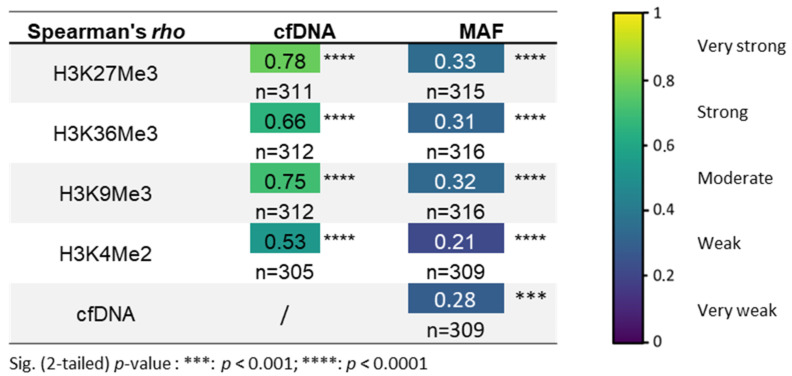
Correlation between methylated-nucleosome levels, cfDNA concentration, and MAF percentage in the global NSCLC cohort at diagnosis. The MAF % was attained via SOPHIA DDM^TM^ bio-informatics analysis, and corresponds to the ratio of the absolute count read between the mutated forms on the reference forms at each genomic position. Spearman’s rank correlation coefficient (Spearman’s rho) was calculated for the combinations represented in the table. *** and **** represent *p*-value < 0.001 and < 0.0001, respectively. The number of samples involved is indicated (*n*) for every correlation. The Spearman’s correlation coefficient range from 0 to 1 is represented on the right panel as a color map.

The circulating nucleosomes containing the methylated histone marks, and the quantity of cfDNA, or the percentage of mutated allele fraction (MAF) correlated well (Figure 2). The training and validation sets were combined into one whole cohort, to increase the number of samples per group, for a better accuracy (n*_NSCLC_* = 319; n*_HEALTHY_* = 201). At diagnosis, patients with NSCLC showed a strong positive correlation between levels of cfDNA and of H3K27Me3, H3K36Me3, or H3K9Me3 nucleosome (*r* = 0.78; 0.66, and 0.75, respectively; *p*-value < 0.0001), and a moderate correlation with the H3K4Me2 nucleosome (*r* = 0.53; *p*-value < 0.0001). The percentage of MAF for genomic somatic alterations in the plasma samples was defined using the ratio of the absolute count read between the reference and mutated forms. All H3K27Me3-, H3K36Me3-, H3K9Me3-, and H3K4Me2-nucleosome levels and the cfDNA concentration showed a weak, but statistically highly significant correlation coefficient with the percentage of MAF (*r* = 0.33, 0.31, 0.32, and 0.21, respectively; *p*-value < 0.0001, and *r* = 0.28; *p*-value < 0.001, respectively) (Figure 2). The strongest correlations were found between the cfDNA and H3K27Me3-nucleosome levels, and between the MAF and H3K27Me3-nucleosome levels.

### 3.3. H3K27Me3 Expression Level in Normal and Adenocarcinoma Tissue

**Figure 3:**
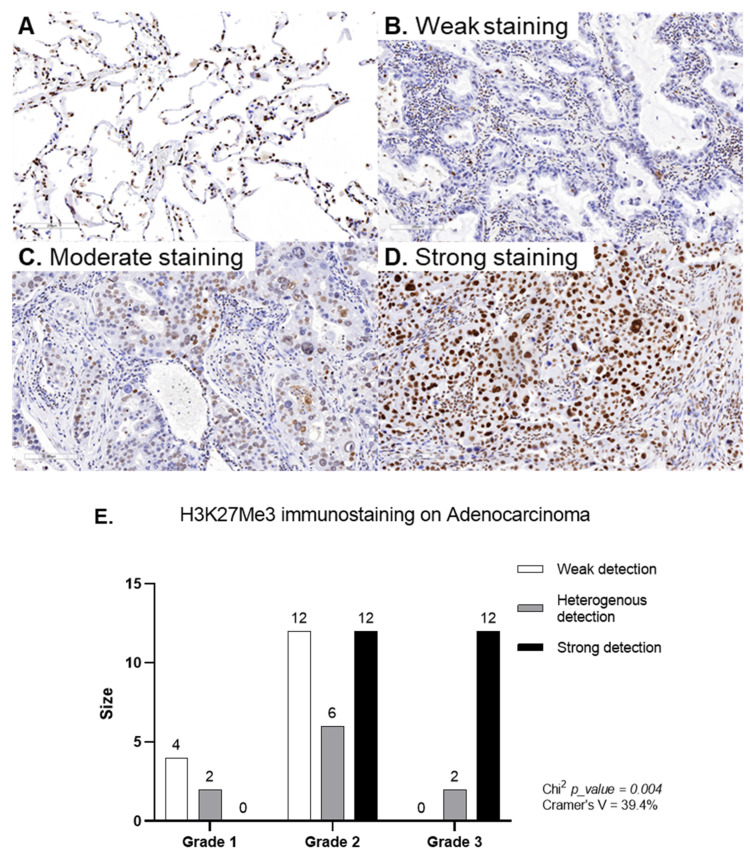
Representative H3K27Me3 immunostaining (10x objective). (**A**) Representative image of normal lung tissue. (**B**–**D**) Representative images of weak- (**B**), moderate- (**C**), and high-intensity (**D**) immunostaining from a lung adenocarcinoma. (**E**) H3K27Me3 expression as a function of the grade.

The immunohistochemical investigation of tissue microarrays (TMAs) in normal pneumocytes (*n* = 18) showed a moderate to high H3K27Me3 expression level in 83% of cases (Figure 3A). In the alveolar region (parenchyma) of normal lung tissue, where air and blood are brought into closed proximity over a large surface, the barrier between the air and blood consists of a continuous alveolar epithelium (a mosaic of type I and type II alveolar epithelial cells), a continuous capillary endothelium, and the connective tissue layer in between, as shown in Figure 3A. In contrast, in the tumoral tissue (*n* = 50, two cores per patient), remodeling structural events occurred (Figure 3B–D). We observed (i) a weak H3K27Me3 expression level in 42% of the cases (Figure 3B), (ii) a moderate expression level in 37% of the cases (Figure 3C), and (iii) a high expression level in 21% of the cases (Figure 3D). The TMAs of lung adenocarcinoma showed heterogeneous H3K27Me3 expression levels within the same patient, demonstrating an intra-tumoral heterogeneity The analysis of H3K27Me3 expression as a function of grade showed that the expression of H3K27Me3 is associated with the tumor grade. A higher H3K27Me3 expression was observed in tumor grades 2 and 3 (Figure 3E). In contrast, the H3K27Me3-expression heterogeneity was not associated with the tumor grade.

### 3.4. Relevant Combined Information from the Circulating H3K27Me3-Nucleosome Levels and ctDNA Molecular Profile at Diagnosis

**Figure 4:**
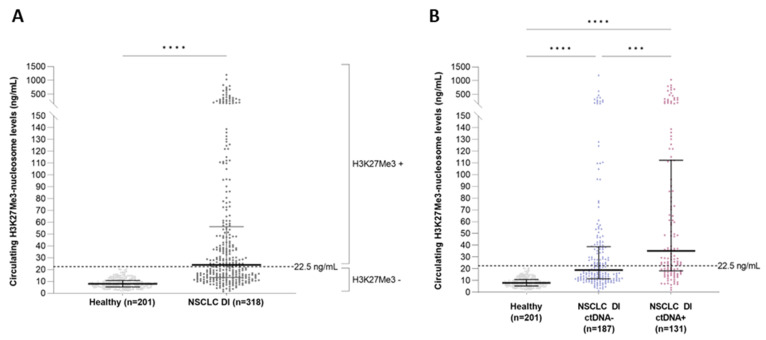
Quantification of circulating H3K27Me3 nucleosomes in NSCLC at diagnosis, and in healthy samples, in whole cohorts. (**A**) Healthy samples (light grey) and NSCLC samples at diagnosis (DI) (dark grey). (**B**) Healthy samples (light grey), NSCLC samples at DI in which somatic mutation was detected (ctDNA+; pink) or not (ctDNA-; blue), according to the somatic molecular profile obtained using a panel of 78 genes, containing the major known oncodrivers. Whiskers represent the 25th–75th percentile with median. *** and **** represent *p*-value < 0.001 and < 0.0001, calculated using the Mann–Whitney (groups of interest (k) = 2) and Kruskal–Wallis (*k* > 2) tests.

As H3K27Me3-nucleosome level was high in the NSCLC samples, but showed a low, although significant, correlation with the MAF; we asked if H3K27Me3 levels could bring additional information, relative to the potential presence of circulating tumor material or epigenetically modified nucleosomes. To address this question, we first defined the upper limit of the reference interval of H3K27Me3 nucleosomes in a healthy population. As the distribution of the training and validation sets was similar (Figure S2), we combined the two sets of samples into a global and homogenous healthy cohort (*n* = 201), in which no outlier was identified using either the D/R ratio or ROUT method. The upper limit of the reference interval, including 95% of this population, was calculated at 18.4 ng/mL (SD = 4.07). Then, we defined a cut off at 22.5 ng/mL (18.4 + 1 SD), ensuring that all the heathy samples were below the cut off in both the training and validation sets. This cut off is largely above the lower limit of detection (LLOQ) of the assay, which was defined at 2.79 ng/mL. In the whole NSCLC cohort (*n* = 318), 53.1% of the patients (*n* = 169) showed H3K27Me3-nucleosome levels above the cut off (Figure 4A). The presence or absence of somatic alterations was reported for the same plasma samples, based on DNAseq next-generation sequencing (NGS) analysis (the comprehensive panel contained the major 78 oncodrivers known in NSCLC). In the whole NSCLC cohort, there were 41.2% of samples in which at least one somatic alteration or copy number variation (CNV) was identified, referred to hereafter as a ctDNA-positive sample (ctDNA+; *n* = 131), and 58.8% of patients for whom no genetic somatic alteration was found, defined as ctDNA-negative (ctDNA-; *n* = 187) hereafter. The level of H3K27Me3 nucleosomes was significantly higher in the ctDNA+ group, compared to the ctDNA- group (median = 33.9 ng/mL vs. 18.5 ng/mL; *p*-value < 0.001), and compared to the healthy group (median = 33.9 ng/mL vs. 8.0 ng/mL; *p*-value < 0.0001) (Figure 4B). The H3K27Me3-nucleosome levels were also higher in the ctDNA- group, compared to the healthy group (median = 18.5 ng/mL vs. 8.0 ng/mL; *p*-value < 0.0001).

**Figure 5:**
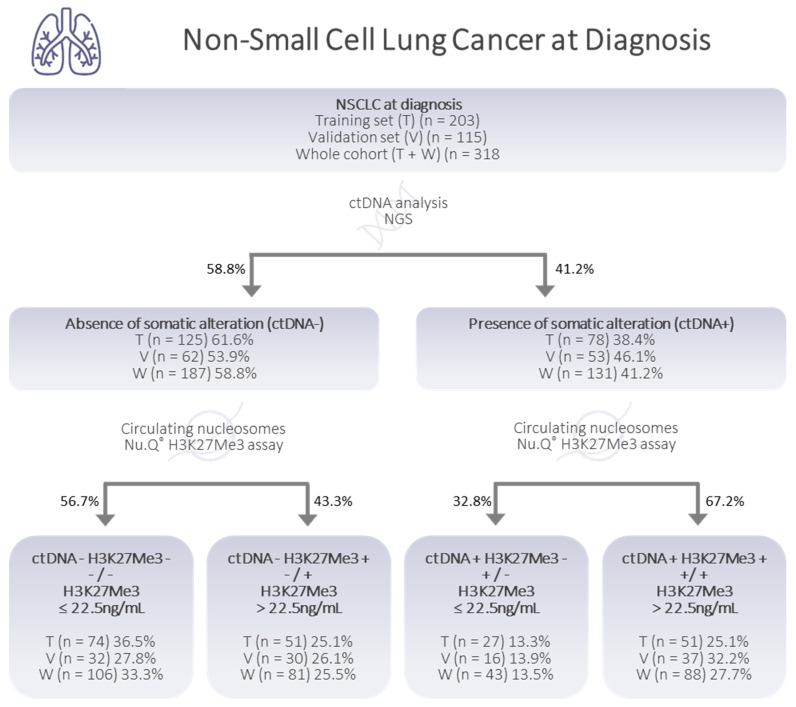
Decision tree proposed for the classification of NSCLC samples at diagnosis. The decision is based on the presence/absence of circulating tumor DNA (ctDNA), and on H3K27Me3-nucleosome levels below or above 22.5 ng/mL, determined via K2-EDTA plasma samples from NSCLC patients at diagnosis. The number of samples (*n*) is presented for the training set (T; *n* = 203), the validation set (V; *n* = 115), and the whole cohort (V + T; *n* = 318). The percentages expressing the part of the total cohort involved are presented in the boxes. The distribution of sub-groups on either side of the decision tree arms is also shown as a percentage.

Based on these observations, we compared the tumor mutation burden defined by the ctDNA status of the samples, and the H3K27Me3-nucleosome concentrations, in a decision tree model (Figure 5). We sub-classified the samples based on their ctDNA status (ctDNA- or ctDNA+), and based on their H3K27Me3-nucleosome levels, defined as H3K27Me3-positive (H3K27Me3+) or H3K27Me3-negative (H3K27Me3-), depending on whether the H3K27Me3-nucleosome levels were above or below the defined cut off, at 22.5 ng/mL. By combining these markers in a decision tree approach, using the results of the DNA analysis (ctDNA- or ctDNA+) as a root node, and then the H3K27Me3-nucleosome levels (H3K27Me3- and H3K27Me3+) as an internal node, we observed that: (i) 33.3% of samples were double-negative for ctDNA and H3K27Me3; (ii) 25.5% of samples were negative for somatic alteration, but had a high level of H3K27Me3 nucleosomes; (iii) 13.5% of samples had a low level of H3K27Me3, even if somatic alterations had been detected; and (iv) 27.7% of samples were found to be positive for both ctDNA and H3K27me3 nucleosomes (Figure 5).

### 3.5. High Level of Circulating H3K27Me3 Nucleosomes Observed in NSCLC Samples during Treatment Is More Pronounced in the Presence of Mutated ctDNA

**Figure 6:**
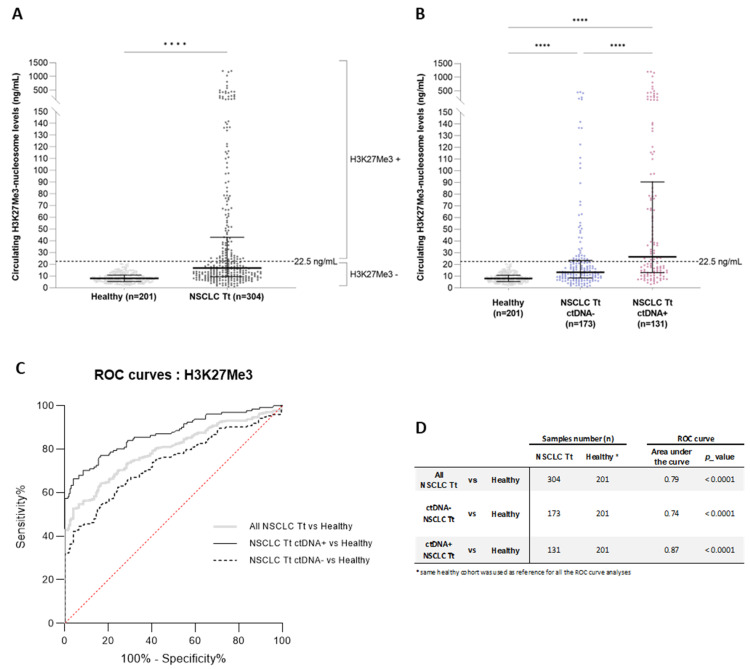
Quantification of circulating H3K27Me3 nucleosomes in NSCLC and healthy samples in the whole cohort during treatment. The concentration of circulating H3K27Me3 nucleosomes was measured using chemiluminescent Nu.Q^®^ immunoassays on human K2-EDTA plasma samples from NSCLC patients during treatment (Tt), and from healthy donors. (**A**,**B**) The jitter plot analyses representing the circulating H3K27Me3-nucleosome levels (**A**) in NSCLC during treatment (light grey), compared with healthy samples (dark grey), or (**B**) in NSCLC samples during treatment in which mutation is detected (ctDNA+; pink) or not (ctDNA-; blue), according to the somatic molecular profile obtained using a panel of 78 genes, containing the major oncodrivers known in NSCLC. The whiskers represent the 25th–75th percentile with median. **** represent *p*-value < 0.0001, calculated using the Mann–Whitney (groups of interest (k) = 2) and Kruskal–Wallis (*k* > 2) tests. (**C**). The receiver-operating characteristic (ROC) curve analysis of circulating H3K27Me3 nucleosomes. The discrimination of the NSCLC during treatment versus the healthy groups (grey line) is compared to the discrimination of NSCLC ctDNA+ during treatment, versus the healthy groups (black line), and to the discrimination of NSCLC ctDNA- during treatment, versus the healthy groups (dotted line). The red line indicates the theoretical random chance. (**D**) The group size, area under the curve (AUC), and corresponding *p*-value for the targeted groups are listed. All analyses are highly significant.

Based on the results observed at diagnosis, we decided to focus our analysis of NSCLC samples collected during patient treatment on H3K27Me3 only, to evaluate its level change across the treatment. We assessed the concentration of circulating H3K27Me3 nucleosomes in NSCLC samples (*n* = 304) in routine settings, and compared them to healthy samples (*n* = 201). A highly significant increase was observed in the NSCLC samples, compared to the healthy samples (median = 16.9 ng/mL vs. 8 ng/mL, respectively; *p*-value < 0.0001) (Figure 6A). In this NSCLC population, the H3K27Me3-nucleosome levels were lower than the samples collected at diagnosis (median_DURING TREATMENT_ = 16.9 ng/mL vs. median_AT DIAGNOSIS_ = 24 ng/mL *p*-value < 0.0001).

The molecular profiles of these NSCLC samples were conducted via NGS analyses. In 43.1% of the samples (*n* = 131), at least one mutation among the 78 screened genes was detected, demonstrating the presence of ctDNA in the plasma samples. Then, we compared the circulating H3K27Me3-nucleosome levels in the positive and negative ctDNA samples (ctDNA+, ctDNA-) collected during the treatment. The level of circulating H3K27Me3 nucleosomes was lower in the ctDNA-negative group, compared to the ctDNA-positive group (median_ctDNA-_ = 13.4 ng/mL vs. median_ctDNA+_ = 26.1 ng/mL, respectively, *p*-value < 0.0001) (Figure 6B). A greater heterogeneity in H3K27Me3-nucleosome levels was observed in the presence of a somatic alteration; 55% of the samples were found above the H3K27Me3-nucleosome cut off in the ctDNA+ group, whereas only 26.6% of the samples were above this cut off in the ctDNA- group (Figure 6B). The clinical performance was then evaluated for the whole cohort (in grey), and for the ctDNA- (dotted line) and ctDNA+ (in black) sub-groups, in comparison to the healthy samples (Figure 6C, Table S3). We found that the discrimination of NSCLC samples was improved in the ctDNA+ sub-group, with an AUC of 0.87, compared to 0.74 for the ctDNA- sub-group, and 0.79 for the whole cohort, respectively; *p*-value < 0.0001) (Figure 6C,D).

### 3.6. Additive Value of Both Biomarkers: H3K27Me3 Nucleosomes and ctDNA

**Figure 7:**
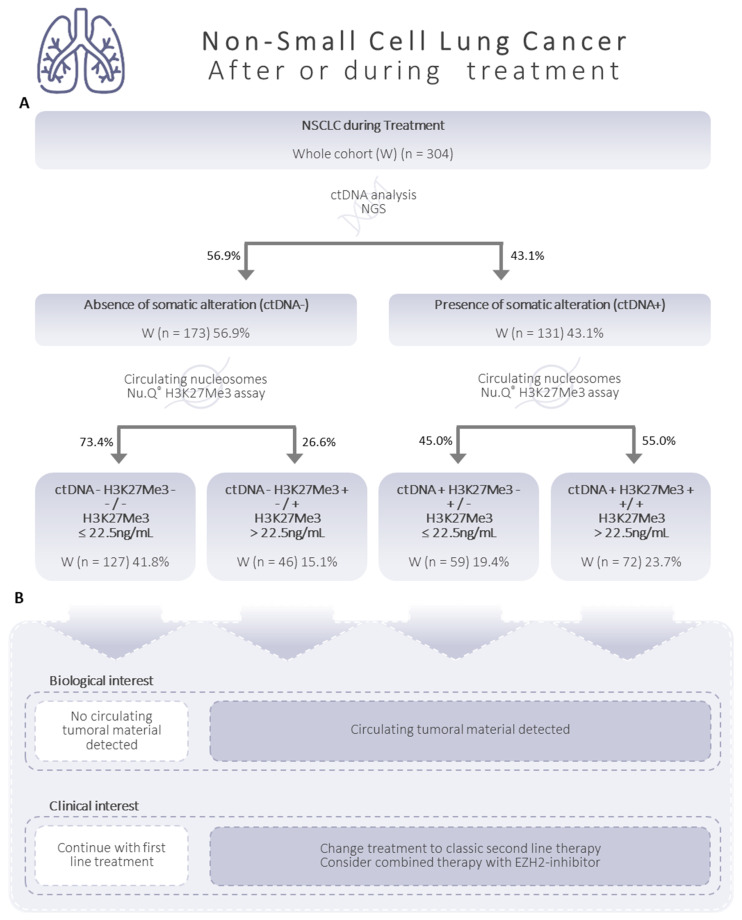
Decision tree proposed for the classification of NSCLC samples during patient follow-up. (**A**) The decision is based on H3K27Me3-nucleosome levels below or above 22.5 ng/mL, and on the presence/absence of circulating tumor DNA (ctDNA), determined using K2-EDTA plasma samples from NSCLC patients during treatment. The number of samples (*n*) is presented for the whole cohort (W; *n* = 304). The percentages expressing the part of the total cohort involved are presented in the boxes. The distribution of sub-groups on either side of the decision tree arms is also shown as a percentage. (**B**) Biological and clinical interests are based on our proper interpretation, and should be validated clinically.

After the sub-grouping of the NSCLC samples collected during treatment according to their ctDNA- and ctDNA+ classification (56.9% and 46.1%, respectively); samples were further classified based on their level of circulating H3K27Me3 nucleosomes below or above the cut off at 22.5 ng/mL, as H3K27Me3-negative (H3K27Me3-) or H3K27Me3-positive (H3K27Me3+). We applied the same decision tree principle as previously described, based on the ctDNA analysis, firstly, and then the level of H3K27Me3 nucleosomes (Figure 7A). We observed (i) 41.8% of samples were double-negative for the ctDNA and circulating H3K27Me3-nucleosome levels (ctDNA- H3K27Me3-); (ii) 15.1% of samples were negative for somatic alteration, but positive for circulating H3K27Me3 nucleosomes (ctDNA- H3K27Me3+); (iii) 19.4% of samples showed a level of circulating H3K27Me3 nucleosomes below 22.5 ng/mL, even when somatic alterations had been detected (ctDNA+ H3K27Me3-); (iv) 23.7% of samples were found to be positive for both ctDNA and circulating H3K27Me3-nucleosome levels (ctDNA+ H3K27Me3+).

### 3.7. High Circulating H3K27Me3-Nucleosome Levels Are Preferentially Associated with TP53 Mutations during Treatment

**Figure 8:**
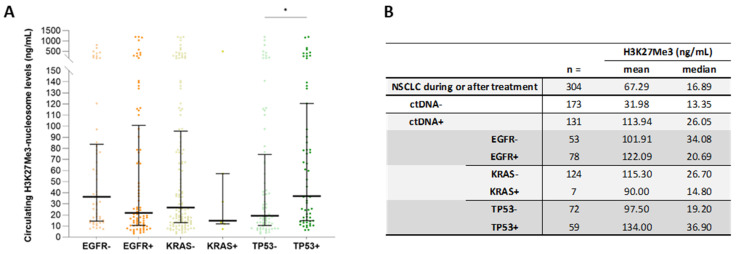
The H3K27Me3-nucleosome levels of the NSCLC samples during treatment, according to the somatic alteration status. The concentration of circulating H3K27Me3 nucleosomes was measured using chemiluminescent Nu.Q^®^ immunoassays on human K2-EDTA plasma samples. Only the ctDNA+ group was included in this analysis. Minus (−) groups referred to samples where a mutation in an oncodriver is different from the gene of interest (*EGFR*, *KRAS*, or *TP53*); plus (+) groups referred to samples in which at least one mutation is detected in the gene of interest (*EGFR*, *KRAS*, or *TP53*). (**A**) the whiskers represent the 25th–75th percentile with median. * represents *p*-value < 0.05 calculated using the Mann–Whitney U test for testing the hypotheses of equality or difference between the two groups of interest. (**B**) The group size (*n*) means and medians are summarized in the table.

In the ctDNA+ group, the levels of circulating H3K27Me3 nucleosomes were compared based on the mutation status of three major oncodrivers of interest: EGFR, KRAS, and TP53. The mutational status in the corresponding sub-groups was annotated as positive (+) when at least one mutation was detected, and negative (−) when no mutation was detected (Figure 7). In parallel with the tendency of association found at diagnosis a significant increase in circulating H3K27Me3-nucleosome levels was observed in the TP53+ (*n* = 72) sub-group, compared to the TP53- (*n* = 59) sub-group (median_TP53+_ = 36.9 ng/mL vs. median_TP53-_ = 19.2 ng/mL; *p*-value < 0.05) (Figure 8A,B). No difference was observed in the comparisons between the EGFR+ versus EGFR-, nor the KRAS+ versus KRAS- sub-groups (median_EGFR+_ = 21.8 ng/mL vs. median_EGFR-_ = 36.3 ng/mL; *p*-value = 0.22; median_KRAS+_ = 14.8 ng/mL vs. median_KRAS-_ = 26.7 ng/mL; *p*-value = 0.50) (Figure 8A,B).

## 4. Discussion

Patient management with NSCLC is based on a histopathological evaluation completed using protein and molecular biomarkers to determine the optimal treatment. Tumor-tissue-based molecular testing has demonstrated its utility in assessing patients for the recommended biomarkers, but remains challenging, due to invasive tissue sampling, the DNA quantity availability and heterogeneity, and the inadequacy of repeated testing [30]. In contrast, the genomic somatic testing of oncodrivers through liquid biopsy has emerged as a key strategy for managing patients that are diagnosed with an advanced NSCLC, and can be used both at diagnosis, to define the first-line therapy, and throughout the patient follow-up, to identify the emergence of resistant mechanisms. Therefore, although tissue biopsy remains the reference standard in cancer diagnosis and patient management, when combined with plasma-based molecular profiling, the patient standard of care is improved by approximately 11 to 20% [31,32,33]. In contrast to tissue biopsies, plasma testing can be easily repeated serially, to monitor the response, and may allow the detection of minimal residual disease prior to a CT scan or clinical progression. It also reduces the invasiveness and costs. Subsequently, it is more compliant for patients. Despite the high clinical specificity of ctDNA, physicians often face a high non-contributive detection rate of preoperative mutated plasma ctDNA, around 24% to 85% in the overall population of lung cancers, associated with the stage, in routine settings [34,35,36]. Even though there have been recent improvements in the sensitivity of sequencing technologies, there are still some limitations to ctDNA-based molecular profiling: (1) technical limitations relating to the low ctDNA concentration, and rare mutation detection; and (2) biological limitations, including tumor shedding [37]. CtDNA was detected after treatment in 64.3% of patients who had obvious clinical recurrence [38]. Moreover, a study conducted by Chaudhuri et al. showed that disease recurrence in the ctDNA-negative population happened in 6% of the post-operative population of NSCLC patients [39]. So, currently, the interpretation of an undetectable ctDNA is still a significant limitation in clinical indication. Other circulating biomarkers may complement ctDNA molecular testing. The circulating nucleosome, as the carrier of genetic and epigenetic information and genetic aberrations, could be one of these. In addition, due to the low volumes required for the nucleosome Nu.Q^®^ immunoassay, it is easy to add it to the plasma molecular profiling testing, using the same plasma sample. In this study, we focused on circulating H3K27Me3-nucleosome levels in NSCLC patients, at diagnosis or during their treatment, and the combined contributive information to the molecular profiling of circulating H3K27Me3 nucleosomes.

TMA analysis showed that the repressive mark H3K27Me3 was detected in the nucleus of normal adjacent cells and adenocarcinoma cells. However, the presence of H3K27Me3 in both types of cells, as with other histone modifications, could be localized in different regions of the genome. Indeed, histone modification is a dynamic process, as demonstrated recently via a FRET (fluorescence resonance energy transfer) biosensor-based technique in cancerous living cells [40]. Moreover, the repression was not randomly distributed within the genome, but enriched in inactive topologically associated domains (TADs) with a low expression of the genes involved in proliferation [41,42]. This illustrates a dynamic distribution of histone marks, both temporally and spatially, inside the nucleus.

As described for genetic clonality profiling and PD-L1 (programmed death ligand 1) tissue expression, we observed the intra- and inter-tumoral heterogeneity in adenocarcinomas [43]. Interestingly, the frequency of a high expression of H3K27Me3 was higher in grade 3 adenocarcinoma, compared to grade 2 and grade 1, suggesting that in NSCLC, like in thyroid cancer or an invasive phenotype of melanoma, H3K27Me3 overexpression could be positively associated with tumor aggressiveness [44,45]. Cellular plasticity and tumor cell dedifferentiation is commonly observed in various types of malignancies, and is described to be associated with an increased tumor cell invasiveness and drug resistance. We could speculate that the association between the H3K27Me3 level and tumor aggressiveness could be linked with its role in this loss of differentiation in tumor cells [46,47,48]. At diagnosis, the circulating H3K27Me3-nucleosome levels are significantly higher in NSCLC patients, compared to healthy donors. In contrast to tissue observation, this analysis of circulating H3K27Me3 nucleosomes reflects the release of nucleosomes into the blood following the cell death and high cell turnover that occur in cancer [8,49,50].

To discuss the advantages of the quantification of H3K27Me3 nucleosomes in clinical settings, we had to establish the normality range into a healthy population, using a similar methodology to that of other tumoral biomarkers, such as CYFRA21.1 and CA125. To assess this, we first determined the upper limit of the reference interval of H3K27Me3 nucleosomes in a healthy population, and determined the threshold of positivity as 22.5 ng/mL, ensuring 100% specificity in both the training and validation sets. The increased H3K27Me3-nucleosome levels observed in NSCLC patients were even more pronounced in patients who have somatic alterations, and were logically positively associated to the cfDNA concentration as their carrier. Somatic mutations were found in 41.2% of the analyzed samples, and 27.7% of the samples were positive for both ctDNA and circulating H3K27Me3 nucleosomes, pointing to putative epigenetic and mutational burden processes. Meanwhile, in 13.5% of samples, only somatic alterations were detected, demonstrating the presence of tumoral material in the blood, but a low level of H3K27Me3 nucleosomes, which might suggest a low-grade tumor or epigenetic changes compared to normal cells, as supported by the TMA analysis performed.

Besides, one quarter of patients (25.5%) had high H3K27Me3-nucleosome levels even if no somatic alteration were detected in the plasma. This is indicative of the tumoral DNA release into the blood, despite the absence of somatic alterations detected, indicating a somatic mutation that is not covered by the comprehensive panel of genes used in the customized NGS assay and, potentially, a high-grade cancer. We can hypothesize that, most likely, the nucleosome and its associated DNA came from cancer cells, but from a genomic region that did not have a cancerous mutation. Those samples could be considered as true ctDNA-negative for the main oncodriver alterations. Nevertheless, we cannot completely exclude the impact of analytical sensitivity of both methods in the detection of the circulating materials. In contrast, 33.3% of the samples were negative for both the ctDNA and H3K27Me3-nucleosome level, indicating no, or very few, tumoral materials released into the blood. In this case, a re-biopsy will probably be required for these patients at diagnosis, until additional cancer biomarkers are identified. Altogether, these results at diagnosis showed that by combining circulating H3K27Me3-nucleosome levels with genomic testing, we can increase the contributing information of ctDNA sequencing by 25.5%, to help interpret negative ctDNA results. In addition, a high level of H3K27Me3 nucleosomes in half of the cases (52%) could be used as a biomarker, to monitor patients during their follow up.

During patients’ treatment, high levels of circulating H3K27Me3 nucleosomes were still observed in NSCLC samples, especially in the presence of mutated ctDNA. Nevertheless, we can note that, despite still being high, the H3K27Me3-nucleosome levels in the ctDNA-negative group were closer to those observed in the healthy group, with 61.2% of the samples below the cut off. As the study was conducted on two independent cohorts, at diagnosis and during treatment, it was not possible to make a direct comparison. However, Holdenrieder and al. described that, in patients with advanced lung cancers, the levels of circulating nucleosomes at diagnosis were significantly lower in patients who responded to chemotherapy [51]. In a recent review, the authors highlighted the measurement of the histones, histone complexes, and histone-associated PTMs as a promising method for discrimination, monitoring, and treatment guiding in solid cancers [52]. Altogether, this suggests that modifications in nucleosomes could be a potential predictive biomarker. When combining ctDNA sequencing and H3K27Me3-nucleosome measurements, we observed that nearly 42% of the samples were negative for both ctDNA and H3K27Me3 nucleosomes, suggesting no detection of tumoral material, and a potential positive evolution of the disease. First-line treatment should be continued, and the next follow up may be spaced out over time (Figure 7B). In 15% of the samples, no somatic mutation was found, but a high level of circulating H3K27Me3 nucleosomes was detected. This could illustrate a progression in the disease in the absence of the acquisition of a known somatic mutation, or no alteration covered by the NGS comprehensive gene panel used, which has a 0.5% sensitivity level. A change in the treatment to classic second-line therapy may be considered (Figure 7B). These results could also suggest that a combined therapy with an EZH2-inhibitor might be appropriate. More clinical data on EZH2 inhibitor performances in NSCLC are required, to confirm or reject this option. Somatic mutations have been detected in the remaining 43% of the samples associated with a high level of H3K27Me3 nucleosomes (23.7%) or not associated (19.4%), demonstrating a progression in the cancer, and the need to change the treatment (Figure 7B). Importantly, when the level of H3K27Me3 nucleosomes was considered with the ctDNA, there was an increased identification of a potential disease progression from 42 to 58% of patients. In addition to the potential benefits of combining H3K27Me3 nucleosomes and ctDNA analysis at diagnosis to ascertain a true negative ctDNA result, we propose that H3K27Me3 nucleosomes could also be a good potential biomarker to detect minimal residual disease, and to monitor the response during the patient follow up. Further studies would be required to validate the potential of circulating H3K27Me3 nucleosomes as a biomarker in NSCLC patient surveillance. When it is validated, we could consider implementing the measurement of H3K27Me3 nucleosomes before molecular profiling analysis, for improved patient care, and a more cost-efficient healthcare system. Indeed, if during post-treatment, the patient is well, with normal scan images and low H3K27Me3-nucleosome levels, the ctDNA analysis could be postponed. In contrast, a high level of H3K27Me3 nucleosomes should trigger a quicker ctDNA analysis and therapeutic decision.

In the French population, the frequency of tissue somatic alterations reported in patients with NSCLC is 11% in *EGFR*, 1% in *HER2*, 29% in *KRAS*, 2% in *BRAF*, and 2% in *PIK3CA* [53]. In our study, the molecular status was not fully associated with the H3K27Me3-nucleosome concentration. Nevertheless, the TP53 status, especially during treatment, seemed to correlate with the H3K27Me3-nucleosome concentration. A total of 40% of TP53 somatic alterations are reported in NSCLC [54,55,56]. This detection is an indicator of a poor prognosis in patients with NSCLC. In advanced NSCLC, patients treated with nivolumab, with or without the CTLA-4 blocker ipilimumab, or pembrolizumab, the median overall survival in the TP53-mutated group was 18.1 months, vs. 8.1 in the TP53-wild-type group. This strongly indicated that TP53 status might be potential predictor of immunotherapy response [57]. These TP53 mutations may differ according to different pathological types and clinical stages, and are often detectable during progression, or in the aggressive grade. These findings agreed with the higher level of H3K27Me3 nucleosome in the TP53 mutated samples observed in our cohorts. In this report, the H3K27Me3-nucleosome concentration was not statistically different according to EGFR and KRAS status, and must be addressed in a wider cohort.

## 5. Conclusions

Using a simple and low-cost H3K27Me3-nucleosome immunoassay to complete the molecular exploration of ctDNA could greatly improve confidence in a negative molecular results at diagnosis. This may allow a reduction in re-biopsy invasive acts. High levels of H3K27Me3 nucleosomes could allow physicians to detect MRD in NSCLC patients at defined intervals of treatment and recovery, to incorporate the analysis of the evolving molecular landscapes during treatment. These data should be confirmed in a longitudinal study, in which NSCLC patients are followed from diagnosis to recovery or relapse.

## 6. Patents

A patent has been applied for, regarding the work reported in this manuscript.

## Data Availability

The data presented in this study are available in the Additional Files, and from the corresponding author: lea.payen-gay@chu-lyon.fr.

## Supplementary Materials

The following supporting information can be downloaded at: https://www.mdpi.com/article/10.3390/biom13081255/s1. Figure S1. (A,B). ROC curve analyses of circulating H3K36Me3 and H3K9Me3 nucleosomes for discrimination in the NSCLC versus healthy groups. The areas under the curve (AUC) for the targeted nucleosome markers are indicated in the legend. The red line indicates the theoretical random chance. All curves were highly significantly different from the chance for circulating H3K36Me3 and H3K9Me3 nucleosomes, and the *p*-values were all < 0.0001 for the training and validation sets. Figure S2. Frequencies distribution of H3K27Me3-nucleosome levels of healthy samples in the training and validation sets. Training and validation sets are represented in white and grey bars, respectively. The *y* axis represents the relative frequency of samples expressed in the percent of the total cohort, along the concentration of H3K27Me3 expressed in ng/mL on the *x* axis. Table S1. Fold change between the levels of methylated nucleosomes in the NSCLC at diagnosis, compared to the healthy samples. The fold change represents a ratio, which is computed as the median level of specific circulating nucleosomes measured in the NSCLC samples, divided by the median level of the reference healthy samples. Table S2. (A,B). Sensitivity and specificity data for the ROC curves in Figure 1 and Figure 6. Data corresponding to Figure 1C and Figure S1A are presented in Table S2A (training set); data corresponding to Figure 6D and Figure S1B are presented in Table S2B (validation set). Table S3. Sensitivity and specificity data for the ROC curve in Figure 6C. Supporting Information S1. Gene panel CAPTURE (chimie IDT) “Tumeur Solide-ADNcir” of the HCL. The custom panel covered 78 genes involved in cancer.

## References

[1] Fan H.C., Blumenfeld Y.J., Chitkara U., Hudgins L., Quake S.R. (2008). Noninvasive diagnosis of fetal aneuploidy by shotgun sequencing DNA from maternal blood. Proc. Natl. Acad. Sci. USA.

[2] Grabuschnig S., Bronkhorst A.J., Holdenrieder S., Rosales Rodriguez I., Schliep K.P., Schwendenwein D., Ungerer V., Sensen C.W. (2020). Putative Origins of Cell-Free DNA in Humans: A Review of Active and Passive Nucleic Acid Release Mechanisms. Int. J. Mol. Sci..

[3] Bronkhorst A.J., Ungerer V., Holdenrieder S. (2019). The emerging role of cell-free DNA as a molecular marker for cancer management. Biomol. Detect. Quantif..

[4] Sanchez C., Roch B., Mazard T., Blache P., Dache Z.A.A., Pastor B., Pisareva E., Tanos R., Thierry A.R. (2021). Circulating nuclear DNA structural features, origins, and complete size profile revealed by fragmentomics. JCI Insight.

[5] Thierry A.R., El Messaoudi S., Gahan P.B., Anker P., Stroun M. (2016). Origins, structures, and functions of circulating DNA in oncology. Cancer Metastasis Rev..

[6] Zhao Z., Shilatifard A. (2019). Epigenetic modifications of histones in cancer. Genome Biol..

[7] Tamkovich S.N., Cherepanova A.V., Kolesnikova E.V., Rykova E.Y., Pyshnyi D.V., Vlassov V.V., Laktionov P.P. (2006). Circulating DNA and DNase activity in human blood. Ann. N. Y. Acad. Sci..

[8] McAnena P., Brown J.A., Kerin M.J. (2017). Circulating Nucleosomes and Nucleosome Modifications as Biomarkers in Cancer. Cancers.

[9] Licht J.D., Bennett R.L. (2021). Leveraging epigenetics to enhance the efficacy of immunotherapy. Clin. Epigenet..

[10] Fenley A.T., Anandakrishnan R., Kidane Y.H., Onufriev A.V. (2018). Modulation of nucleosomal DNA accessibility via charge-altering post-translational modifications in histone core. Epigenet. Chromatin.

[11] Greer E.L., Shi Y. (2012). Histone methylation: A dynamic mark in health, disease and inheritance. Nat. Rev. Genet..

[12] Varambally S., Dhanasekaran S.M., Zhou M., Barrette T.R., Kumar-Sinha C., Sanda M.G., Ghosh D., Pienta K.J., Sewalt R.G., Otte A.P. (2002). The polycomb group protein EZH2 is involved in progression of prostate cancer. Nature.

[13] Comet I., Riising E.M., Leblanc B., Helin K. (2016). Maintaining cell identity: PRC2-mediated regulation of transcription and cancer. Nat. Rev. Cancer.

[14] Kleer C.G., Cao Q., Varambally S., Shen R., Ota I., Tomlins S.A., Ghosh D., Sewalt R.G., Otte A.P., Hayes D.F. (2003). EZH2 is a marker of aggressive breast cancer and promotes neoplastic transformation of breast epithelial cells. Proc. Natl. Acad. Sci. USA.

[15] Wiles E.T., Selker E.U. (2017). H3K27 methylation: A promiscuous repressive chromatin mark. Curr. Opin. Genet. Dev..

[16] Wan L., Li X., Shen H., Bai X. (2013). Quantitative analysis of EZH2 expression and its correlations with lung cancer patients’ clinical pathological characteristics. Clin. Transl. Oncol..

[17] Sato T., Kaneda A., Tsuji S., Isagawa T., Yamamoto S., Fujita T., Yamanaka R., Tanaka Y., Nukiwa T., Marquez V.E. (2013). PRC2 overexpression and PRC2-target gene repression relating to poorer prognosis in small cell lung cancer. Sci. Rep..

[18] Cardenas H., Zhao J., Vieth E., Nephew K.P., Matei D. (2016). EZH2 inhibition promotes epithelial-to-mesenchymal transition in ovarian cancer cells. Oncotarget.

[19] Ma J., Zhang J., Weng Y.C., Wang J.C. (2018). EZH2-Mediated microRNA-139-5p Regulates Epithelial-Mesenchymal Transition and Lymph Node Metastasis of Pancreatic Cancer. Mol. Cells.

[20] Lachat C., Bruyere D., Etcheverry A., Aubry M., Mosser J., Warda W., Herfs M., Hendrick E., Ferrand C., Borg C. (2020). EZH2 and KDM6B Expressions Are Associated with Specific Epigenetic Signatures during EMT in Non Small Cell Lung Carcinomas. Cancers.

[21] Lu C., Han H.D., Mangala L.S., Ali-Fehmi R., Newton C.S., Ozbun L., Armaiz-Pena G.N., Hu W., Stone R.L., Munkarah A. (2010). Regulation of tumor angiogenesis by EZH2. Cancer Cell.

[22] Liu X., Lu X., Zhen F., Jin S., Yu T., Zhu Q., Wang W., Xu K., Yao J., Guo R. (2019). LINC00665 Induces Acquired Resistance to Gefitinib through Recruiting EZH2 and Activating PI3K/AKT Pathway in NSCLC. Mol. Ther. Nucleic Acids.

[23] Passaro A., Janne P.A., Mok T., Peters S. (2021). Overcoming therapy resistance in EGFR-mutant lung cancer. Nat. Cancer.

[24] Gong H., Li Y., Yuan Y., Li W., Zhang H., Zhang Z., Shi R., Liu M., Liu C., Chen C. (2020). EZH2 inhibitors reverse resistance to gefitinib in primary EGFR wild-type lung cancer cells. BMC Cancer.

[25] Duan R., Du W., Guo W. (2020). EZH2: A novel target for cancer treatment. J. Hematol. Oncol..

[26] Garcia J., Dusserre E., Cheynet V., Bringuier P.P., Brengle-Pesce K., Wozny A.S., Rodriguez-Lafrasse C., Freyer G., Brevet M., Payen L. (2017). Evaluation of pre-analytical conditions and comparison of the performance of several digital PCR assays for the detection of major EGFR mutations in circulating DNA from non-small cell lung cancers: The CIRCAN_0 study. Oncotarget.

[27] Bieler J., Pozzorini C., Garcia J., Tuck A.C., Macheret M., Willig A., Couraud S., Xing X., Menu P., Steinmetz L.M. (2021). High-Throughput Nucleotide Resolution Predictions of Assay Limitations Increase the Reliability and Concordance of Clinical Tests. JCO Clin. Cancer Inform..

[28] Garcia J., Gauthier A., Lescuyer G., Barthelemy D., Geiguer F., Balandier J., Edelstein D.L., Jones F.S., Holtrup F., Duruisseau M. (2021). Routine Molecular Screening of Patients with Advanced Non-SmallCell Lung Cancer in Circulating Cell-Free DNA at Diagnosis and During Progression Using OncoBEAM(TM) EGFR V2 and NGS Technologies. Mol. Diagn. Ther..

[29] Garcia J., Kamps-Hughes N., Geiguer F., Couraud S., Sarver B., Payen L., Ionescu-Zanetti C. (2021). Sensitivity, specificity, and accuracy of a liquid biopsy approach utilizing molecular amplification pools. Sci. Rep..

[30] Pennell N.A., Arcila M.E., Gandara D.R., West H. (2019). Biomarker Testing for Patients With Advanced Non-Small Cell Lung Cancer: Real-World Issues and Tough Choices. Am. Soc. Clin. Oncol. Educ. Book.

[31] Leighl N.B., Page R.D., Raymond V.M., Daniel D.B., Divers S.G., Reckamp K.L., Villalona-Calero M.A., Dix D., Odegaard J.I., Lanman R.B. (2019). Clinical Utility of Comprehensive Cell-free DNA Analysis to Identify Genomic Biomarkers in Patients with Newly Diagnosed Metastatic Non-small Cell Lung Cancer. Clin. Cancer Res..

[32] Mack P.C., Banks K.C., Espenschied C.R., Burich R.A., Zill O.A., Lee C.E., Riess J.W., Mortimer S.A., Talasaz A., Lanman R.B. (2020). Spectrum of driver mutations and clinical impact of circulating tumor DNA analysis in non-small cell lung cancer: Analysis of over 8000 cases. Cancer.

[33] Cui W., Milner-Watts C., McVeigh T.P., Minchom A., Bholse J., Davidson M., Yousaf N., MacMahon S., Mugalaasi H., Gunapala R. (2022). A pilot of Blood-First diagnostic cell free DNA (cfDNA) next generation sequencing (NGS) in patients with suspected advanced lung cancer. Lung Cancer.

[34] Kuang P.P., Li N., Liu Z., Sun T.Y., Wang S.Q., Hu J., Ou W., Wang S.Y. (2020). Circulating Tumor DNA Analyses as a Potential Marker of Recurrence and Effectiveness of Adjuvant Chemotherapy for Resected Non-Small-Cell Lung Cancer. Front. Oncol..

[35] Nagasaka M., Uddin M.H., Al-Hallak M.N., Rahman S., Balasubramanian S., Sukari A., Azmi A.S. (2021). Liquid biopsy for therapy monitoring in early-stage non-small cell lung cancer. Mol. Cancer.

[36] Markou A.N., Londra D., Stergiopoulou D., Vamvakaris I., Potaris K., Pateras I.S., Kotsakis A., Georgoulias V., Lianidou E. (2023). Preoperative Mutational Analysis of Circulating Tumor Cells (CTCs) and Plasma-cfDNA Provides Complementary Information for Early Prediction of Relapse: A Pilot Study in Early-Stage Non-Small Cell Lung Cancer. Cancers.

[37] Chan H.T., Chin Y.M., Low S.K. (2022). Circulating Tumor DNA-Based Genomic Profiling Assays in Adult Solid Tumors for Precision Oncology: Recent Advancements and Future Challenges. Cancers.

[38] Gale D., Heider K., Ruiz-Valdepenas A., Hackinger S., Perry M., Marsico G., Rundell V., Wulff J., Sharma G., Knock H. (2022). Residual ctDNA after treatment predicts early relapse in patients with early-stage non-small cell lung cancer. Ann. Oncol..

[39] Chaudhuri A.A., Chabon J.J., Lovejoy A.F., Newman A.M., Stehr H., Azad T.D., Khodadoust M.S., Esfahani M.S., Liu C.L., Zhou L. (2017). Early Detection of Molecular Residual Disease in Localized Lung Cancer by Circulating Tumor DNA Profiling. Cancer Discov..

[40] Gong Y., Wei C., Cheng L., Ma F., Lu S., Peng Q., Liu L., Wang Y. (2021). Tracking the Dynamic Histone Methylation of H3K27 in Live Cancer Cells. ACS Sens..

[41] Donaldson-Collier M.C., Sungalee S., Zufferey M., Tavernari D., Katanayeva N., Battistello E., Mina M., Douglass K.M., Rey T., Raynaud F. (2019). EZH2 oncogenic mutations drive epigenetic, transcriptional, and structural changes within chromatin domains. Nat. Genet..

[42] Cai M.Y., Tong Z.T., Zhu W., Wen Z.Z., Rao H.L., Kong L.L., Guan X.Y., Kung H.F., Zeng Y.X., Xie D. (2011). H3K27me3 protein is a promising predictive biomarker of patients’ survival and chemoradioresistance in human nasopharyngeal carcinoma. Mol. Med..

[43] Haragan A., Field J.K., Davies M.P.A., Escriu C., Gruver A., Gosney J.R. (2019). Heterogeneity of PD-L1 expression in non-small cell lung cancer: Implications for specimen sampling in predicting treatment response. Lung Cancer.

[44] Tsai C.C., Chien M.N., Chang Y.C., Lee J.J., Dai S.H., Cheng S.P. (2019). Overexpression of Histone H3 Lysine 27 Trimethylation Is Associated with Aggressiveness and Dedifferentiation of Thyroid Cancer. Endocr. Pathol..

[45] Hoffmann F., Niebel D., Aymans P., Ferring-Schmitt S., Dietrich D., Landsberg J. (2020). H3K27me3 and EZH2 expression in melanoma: Relevance for melanoma progression and response to immune checkpoint blockade. Clin. Epigenet..

[46] Holzel M., Bovier A., Tuting T. (2013). Plasticity of tumour and immune cells: A source of heterogeneity and a cause for therapy resistance?. Nat. Rev. Cancer.

[47] Yuan S., Norgard R.J., Stanger B.Z. (2019). Cellular Plasticity in Cancer. Cancer Discov..

[48] Li J., Stanger B.Z. (2020). How Tumor Cell Dedifferentiation Drives Immune Evasion and Resistance to Immunotherapy. Cancer Res..

[49] Holdenrieder S., Nagel D., Schalhorn A., Heinemann V., Wilkowski R., von Pawel J., Raith H., Feldmann K., Kremer A.E., Muller S. (2008). Clinical relevance of circulating nucleosomes in cancer. Ann. N. Y. Acad. Sci..

[50] Schwarzenbach H., Hoon D.S., Pantel K. (2011). Cell-free nucleic acids as biomarkers in cancer patients. Nat. Rev. Cancer.

[51] Holdenrieder S., Stieber P., von Pawel J., Raith H., Nagel D., Feldmann K., Seidel D. (2004). Circulating nucleosomes predict the response to chemotherapy in patients with advanced non-small cell lung cancer. Clin. Cancer Res..

[52] Tsoneva D.K., Ivanov M.N., Conev N.V., Manev R., Stoyanov D.S., Vinciguerra M. (2023). Circulating Histones to Detect and Monitor the Progression of Cancer. Int. J. Mol. Sci..

[53] Barlesi F., Mazieres J., Merlio J.P., Debieuvre D., Mosser J., Lena H., Ouafik L., Besse B., Rouquette I., Westeel V. (2016). Routine molecular profiling of patients with advanced non-small-cell lung cancer: Results of a 1-year nationwide programme of the French Cooperative Thoracic Intergroup (IFCT). Lancet.

[54] Mitsudomi T., Hamajima N., Ogawa M., Takahashi T. (2000). Prognostic significance of p53 alterations in patients with non-small cell lung cancer: A meta-analysis. Clin. Cancer Res..

[55] Gu J., Zhou Y., Huang L., Ou W., Wu J., Li S., Xu J., Feng J., Liu B. (2016). TP53 mutation is associated with a poor clinical outcome for non-small cell lung cancer: Evidence from a meta-analysis. Mol. Clin. Oncol..

[56] Guo Y., Song J., Wang Y., Huang L., Sun L., Zhao J., Zhang S., Jing W., Ma J., Han C. (2020). Concurrent Genetic Alterations and Other Biomarkers Predict Treatment Efficacy of EGFR-TKIs in EGFR-Mutant Non-Small Cell Lung Cancer: A Review. Front. Oncol..

[57] Assoun S., Theou-Anton N., Nguenang M., Cazes A., Danel C., Abbar B., Pluvy J., Gounant V., Khalil A., Namour C. (2019). Association of TP53 mutations with response and longer survival under immune checkpoint inhibitors in advanced non-small-cell lung cancer. Lung Cancer.

