# Supplementary figures and images for: Circulating H3K27 Methylated Nucleosome Plasma Concentration: Synergistic Information with Circulating Tumor DNA Molecular Profiling
Source: Biomolecules. 2023 Aug 16;13(8):1255. doi: 10.3390/biom13081255 (PMC10452235; doi:10.3390/biom13081255)

## ADDITIONAL FIGURES

Figure S1. A-B.

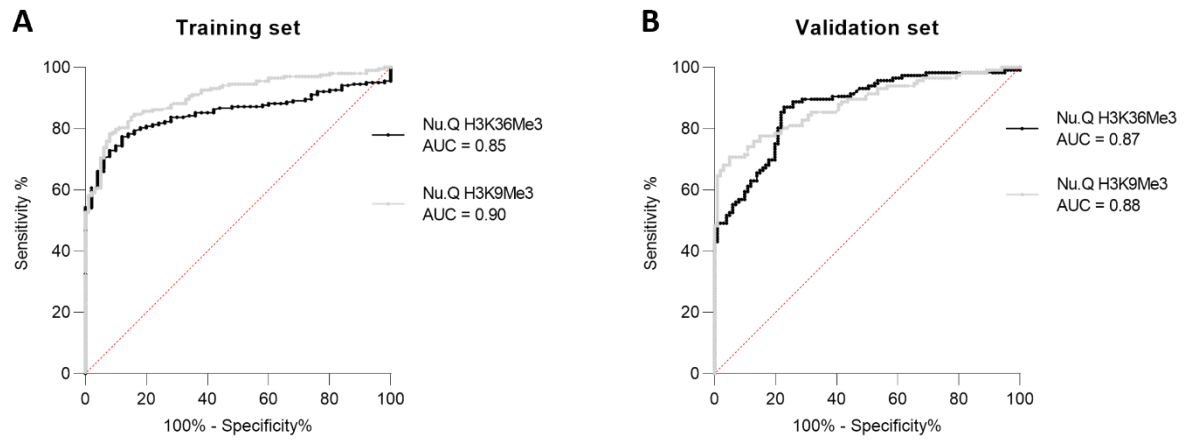

Figure S2.

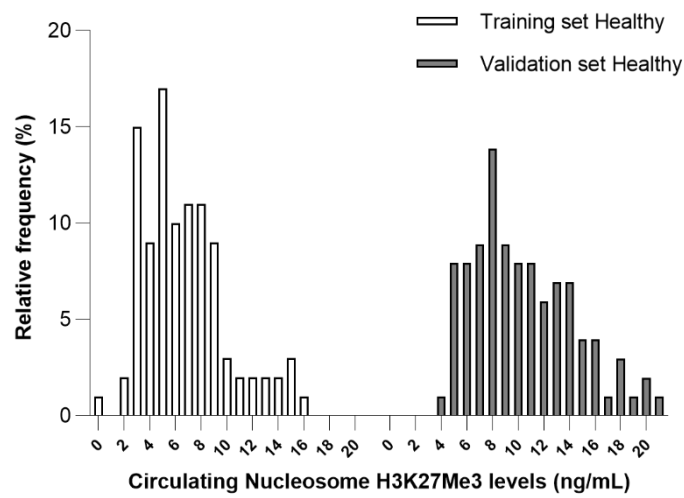

Supplement: Supplementary file 1 [file biomolecules-13-01255-s001.zip › GROLLEAU et al_Biomolecules_Additional Figures.pdf]
